# A search for synergy in the binding kinetics of Trastuzumab and Pertuzumab whole and F(ab) to Her2

**DOI:** 10.1038/npjbcancer.2015.12

**Published:** 2015-08-05

**Authors:** Wai-Heng Lua, Samuel Ken-En Gan, David Philip Lane, Chandra Shekhar Verma

**Affiliations:** 1 Bioinformatics Institute, Agency for Science, Technology, and Research (A*STAR), Singapore, Singapore; 2 p53 Laboratory, Agency for Science, Technology, and Research (A*STAR), Singapore, Singapore; 3 Department of Biological Sciences, National University of Singapore (NUS), Singapore, Singapore; 4 School of Biological Sciences, Nanyang Technological University (NTU), Singapore, Singapore

## Abstract

Therapeutic efficacy resulting from combining Trastuzumab and Pertuzumab in the treatment of Her2 overexpressing breast cancer patients has been shown to increase patient survival. This is thought to arise from inhibition of receptor dimerization and the immune tagging of the cancer cells; however, the underlying molecular mechanisms have remained enigmatic. Previously, a molecular modeling study suggested that this resulted from colocalization of the two antibodies on to the extracellular domain of Her2. We report here the experimental characterization of this interaction by measuring the binding kinetics of these two whole antibodies and their F(ab)s to the extracellular domain of Her2 in solution. We found that both antibodies (the whole antibodies and the fragments) colocalized on to Her2, but did not augment the binding of each other.

## Correspondence/Findings

**Figure 1:**
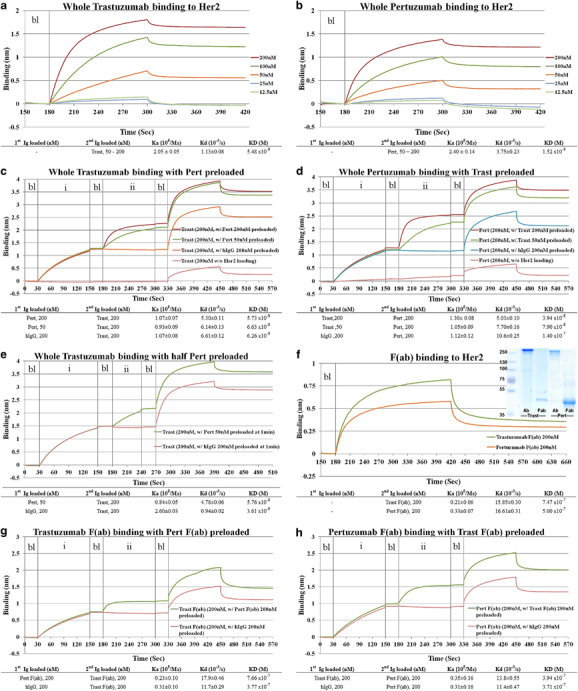
Binding assays of Trastuzumab and Pertuzumab to Her2 on a BLItz system. (**a**) Whole Trastuzumab binding to Her2. Binding of 12.5–200 nM of Trastuzumab to 25 μg/ml of Ni-NTA probe bound Her2-His-tagged on the BLItz system. The binding kinetics of Trastuzumab was titratable to 25 nM. (**b**) Whole Pertuzumab binding to Her2. Binding of 12.5–200 nM of Pertuzumab to 25 μg/ml of Ni-NTA probe bound Her2-His-tagged on the BLItz system. The binding kinetics of Pertuzumab was titratable to 25 nM. (**c**) Whole Trastuzumab binding with Pert preloaded. Binding of 200 nM Trastuzumab to (i) Her2-His-tagged (ii) 50 and 200 nM Pertuzumab or 200 nM human IgG. Unbound sensor was used as negative control. (**d**) Whole Pertuzumab binding with Trast preloaded. Binding of 200 nM Pertuzumab to (i) Her2-His-tagged (ii) 50 and 200 nM Trastuzumab or 200 nM human IgG. Unbound sensor was used as negative control. (**e**) Whole Trastuzumab binding with half Pert preloaded. Binding of 200 nM Trastuzumab to (i) Her2-His-tagged (ii) 50 nM Pertuzumab or 200 nM human IgG at 1 min loading time. One  minute loading time was used to prevent saturation of Pertuzumab, which may interfere with Trastuzumab binding. (**f**) F(ab) binding to Her2. Binding assay showing 200 nM of Trastuzumab or Pertuzumab F(ab) to Her2-His-tagged. Decreased maximum binding kinetics reflect the decreased bound protein size. (top left) SDS-PAGE showing purified Trastuzumab and Pertuzumab F(ab) prepared using the Fab preparation kit. (**g**) Trastuzumab F(ab) binding with Pert F(ab) preloaded. Binding of 200 nM Trastuzumab F(ab) to (i) Her2-His-tagged (ii) 200 nM of Pertuzumab F(ab) or human IgG. (**h**) Pertuzumab F(ab) binding with Trast F(ab) preloaded. Binding of 200 nM Pertuzumab F(ab) to (i) Her2-His-tagged (ii) 200 nM of Trastuzumab F(ab) or human IgG. Bl, baseline, as measured using phosphate-buffered saline; hIgG, human IgG control; Ni-NTA, Nickel-nitrilotriacetic acid; Pert, Pertuzumab; Trast, Trastuzumab.

Therapeutic efficacy resulting from combining drugs in oncology is increasingly being reported. A significant improvement in survival was recently reported in the treatment of Her2 overexpressing breast cancer patients from combining the antibodies Trastuzumab and Pertuzumab.^1^ However, the underlying molecular mechanisms have remained enigmatic. A hypothesis was put forward from a molecular modeling study that suggested a partial rationale in the form of enhanced affinities arising from colocalization of the two antibodies on to the extracellular domain of Her2.^2^ We report here the experimental characterization of this interaction by measuring the binding kinetics (using the BLItz biosensor system, ForteBio, Pall, Singapore) of these two antibodies (whole antibodies and F(ab)s; see Figure 1) to the extracellular domain of Her2 in solution.

Briefly, 25 μg/ml of HIS-tagged Her2 (cat no: 10004-H08H, Sino Biologicals, China) was bound onto Nickel-nitrilotriacetic acid (Ni-NTA) biosensors (ForteBio, Pall). Trastuzumab (Roche, Singapore) and Pertuzumab (Roche) to Her2 binding kinetics were measured using the BLI technology from ForteBio (http://www.fortebio.com/bli-technology.html). Whole antibody-Her2 interactions were calculated from five serial 1:2 dilutions. Synergistic binding of the whole antibodies and F(ab)s were measured from successive loading of the antibodies. Human IgG control (cat no: PN 18-1073, lot no: 3060036, ForteBio, Pall) was used as the control IgG. The F(ab)s of both Trastuzumab and Pertuzumab were prepared by papain digestion using the Pierce^™^ Fab Preparation Kit (cat no: 44985, Life Technologies, Singapore).

Our measurements found the kinetics of whole Trastuzumab to be comparable to previous surface plasmon resonance measurements.^3^ We see that Trastuzumab (in both its IgG and F(ab) states) (see Figure 1a,b,f, respectively) binds tighter than the corresponding Pertuzumab states, an observation in support of the earlier computational model based on the F(ab) states.^2^ Preloading of Her2 with saturating (200 nM) and non-saturating (50 nM) concentrations of each antibody (shown in plateau sections of Figure 1c–e) followed by measurements of the second antibody appears to have little effect on the binding of the latter. An observation to note is that the presence of the control IgG does seem to increase the Kd of both antibodies (compare Figure 1a, c and Figure 1b, d) thus making comparisons with binding of antibodies alone, a little complex.

We repeated the experiments to rule out confounding variables under the following conditions: (1) using only Trastuzumab and Pertuzumab F(ab)s (Figure 1g, h); (2) preloading Her2 with F(ab) or whole antibody, followed by measurement of the alternative antibody as a whole or F(ab), respectively (not shown); (3) preloading the alternative antibody overnight (performed for both F(ab)s and whole antibodies, data not shown). Under these conditions, minimal binding changes of either antibody were observed. This suggests that the state of the antibodies (F(ab)s or whole Igs, or mixed) were not contributing factors nor was the time of incubation a factor in inducing synergistic binding.

We have demonstrated, in agreement with the computational modeling, that Tratsuzumab binds to the extracellular domain of Her2 with a higher affinity than does Pertuzumab. We have also shown, in agreement with the modeling, that both antibodies can colocalize onto the extracellular domain of Her2. However, in contrast to the modeling, we did not observe significant synergistic binding effects through prior Trastuzumab or Pertuzumab loading (note that only the extracellular portion of Her2 was used in our experiments). The discrepancy between these findings and the hypotheses generated from the modelling^2^ may arise from the fact that the modeling may not have captured the complex nature of the interactions which, owing to the much larger system size of the ternary complexes of Her2–Trastuzumab–Pertuzumab, would likely require longer simulation times than that have been used. However, the complex nature of these interactions and the vast differences between *in silico, in vitro,* and *in vivo* measurements^4,5^ rule out any unambiguous conclusions. Nevertheless, the simple fact that both antibodies can bind simultaneously and without significant interference, may be sufficient to further impede Her2 dimerization and enforce the engagement of downstream immune effectors to explain the clinical observations.
